# Object-based neglect in number processing

**DOI:** 10.1186/1744-9081-9-5

**Published:** 2013-01-23

**Authors:** Elise Klein, Korbinian Moeller, Daniela Zinsberger, Harald Zauner, Guilherme Wood, Klaus Willmes, Christine Haider, Alfred Gassner, Hans-Christoph Nuerk

**Affiliations:** 1 Section Neuropsychology, Department of Neurology, University Hospital, RWTH Aachen University, Aachen, Germany; 2 IWM-KMRC Knowledge Media Research Center, Tuebingen, Germany; 3 Department of Psychology, Eberhard Karls University, Tuebingen, Germany; 4 BBRZ Group, Neuronetzwerk, Linz, Austria; 5 Department of Psychology, Paris Lodron University, Salzburg, Austria; 6 SKA – Rehabilitation Center of the PVA Großgmain, Department of Neurorehabilitation, Großgmain, Austria; 7 Department of Psychology, Karl Franzens University, Graz, Austria

**Keywords:** Number processing, Number magnitude comparison, Object-based neglect, Multi-digit numbers

## Abstract

Recent evidence suggests that neglect patients seem to have particular problems representing relatively smaller numbers corresponding to the left part of the mental number line. However, while this indicates space-based neglect for representational number space little is known about whether and - if so - how object-based neglect influences number processing.

To evaluate influences of object-based neglect in numerical cognition, a group of neglect patients and two control groups had to compare two-digit numbers to an internally represented standard. Conceptualizing two-digit numbers as objects of which the left part (i.e., the tens digit should be specifically neglected) we were able to evaluate object-based neglect for number magnitude processing.

Object-based neglect was indicated by a larger unit-decade compatibility effect actually reflecting impaired processing of the leftward tens digits. Additionally, faster processing of within- as compared to between-decade items provided further evidence suggesting particular difficulties in integrating tens and units into the place-value structure of the Arabic number system.

In summary, the present study indicates that, in addition to the spatial representation of number magnitude, also the processing of place-value information of multi-digit numbers seems specifically impaired in neglect patients.

## Introduction

Hemi-spatial neglect typically follows focal brain lesions in the parietal cortex [1] or regions interconnected with the parietal cortex [2,3]. Patients suffering from neglect usually fail to explore the side of space contralateral to their brain lesion site ([4]; for a review see [5]). These spatial manifestations of neglect can also be observed in patients’ everyday behaviour. For instance, patients with left-sided neglect may fail to eat food from the left side of their plate, ignore someone who approaches from their left side or fail to reach for an object on their left [6]. When asked to bisect a physical line, neglect patients tend to indicate the midpoint of the horizontal line to be located to the right of the veridical center [7], as if they partially ignored the left part of the line. Thereby, line bisection is a simple and common clinical assessment tool for leftward hemi-spatial neglect, which can be used to quantify neglect severity.

In the current study we investigated the number magnitude representation in patients with unilateral leftward neglect. Examining neglect patients is informative about the neurocognition of numbers in general because of the very specific influence of neglect symptoms on the representation of number magnitude. Typically, neglect patients fail to explore stimuli in the contra-lesional (most often left) side of space (see above). Additionally, the metaphor of a left-to-right oriented mental number line (henceforth MNL) is a widely agreed upon conceptualization of human representation of number magnitude (cf. [8-10]). Combining these two approaches, there is evidence suggesting that number magnitude representation in neglect patients may be particularly problematic for the representation of relatively smaller numbers, corresponding to the left part of the MNL (e.g., [11], see [12] for a review, but see also [13] for a review on dissociations between neglect in visual and numerical space). In the seminal study by Zorzi and colleagues [11], patients with leftward neglect were asked to indicate the midpoint of an orally presented number interval without performing mental calculations. The authors reported a significant rightward displacement of the midpoints reported by the neglect patients (e.g., reporting 7 to be the middle between 1 and 9). Thereby, the error pattern for mental number line bisection was virtually identical to the performance pattern, which neglect patients exhibit in physical line bisection (e.g., [7]). Additionally, as reported by Halligan and Marshall [14] for the case of line bisection, this rightward displacement in number intervals was directly proportional to the width of the to-be-bisected interval: The wider the segment, the more pronounced the rightward displacement.

The displacement of the midpoint to the right exhibited by neglect patients in number interval bisection is usually interpreted to indicate that number magnitude is represented spatially along a mental number line mimicking physical attributes. From the similar effects of neglect on line bisection and bisection of numerical intervals Zorzi et al., [11] concluded that physical and number space are isomorphic in the sense that they are organised according to similar Cartesian coordinates, which are explored by similar and interacting spatial processing mechanisms (such as the MNL) but which are nevertheless dissociable in principle (see [15]). Yet, it is important to emphasize that the neglect patients exhibiting this form of space-based neglect had intact numerical and arithmetical skills. These original findings of Zorzi et al., [11] were replicated and extended in a number of subsequent studies, employing number interval bisection tasks (e.g., [16-19]) but also other tasks tapping on the magnitude representation of numbers (e.g., [19-21]). Furthermore, the dissociation between impaired explicit processing and spared implicit processing of number magnitude information corroborates the notion that neglect produces a deficit in accessing an intact MNL, rather than a distortion in the representation of the MNL itself [18].

Taken together, there is converging evidence that neglect patients have specific problems to represent relatively smaller numbers corresponding to the left part of the MNL and in particular so when the task at hand requires explicit processing of number magnitude information. However, recent accounts have claimed that number-processing deficits in right-hemisphere-damaged patients may not be due to neglect, but rather due to a working memory deficit (e.g., [22,23]). In particular, it is argued that diverging performance in unilaterally brain damaged patients on tasks such as the number interval bisection task may not necessarily be the consequence of impairments in a spatial-attentional mechanism operating upon a mental number line (isomorphic to physical space), but may be due to an impaired working memory system with a position-specific deficiency. Alternatively, it has been put forward that the deficits in number processing may be due to the fact that the right hemisphere is specialised in processing "small" numbers [24].

However, there are at least two important limitations. First, previous studies have mostly focused on single-digit numbers or rather small numbers in the two-digit number range (see [12] for a review). Therefore, knowledge about the way the representation of multi-digit numbers is affected in patients with neglect is still rather patchy. Second, the vast majority of previous results on the influence of neglect on number processing focused on the phenomenon of space-based conceptualizations of neglect, despite the fact that there is also empirical evidence suggesting other conceptualizations such as object-based symptoms in neglect. In particular, patients with object-based neglect tend to omit the left half of objects displayed across a scene [25-27], whereas patients with spatial neglect can produce an adequate representation of the right half of a scene presented whilst leaving out figures on the left side. Thereby, object-based neglect poses problems for the analysis of the parts of an object as they contribute to the recognition of the whole [28,29], while space-based neglect is concerned with the position of an object as a whole, relative to a predefined spatial reference (e.g., the point of fixation, the subject’s midsagittal plane, or another object in the visual field). One of the first examples of object-based neglect was reported in a study of drawing by Gainotti and colleagues [30]. The authors asked their patients to copy a model containing objects located on a horizontal axis (amongst others a house, a fence, trees). Some patients failed to draw the contralateral side of some of the objects from the model. But even though these patients omitted the left side of some objects they continued to reproduce other objects located even further to the left of the first drawing copies (see also [25,31] for further cases).

### Object based neglect in numerical cognition

As outlined above, neglect can also affect processing of the contra-lesional (mostly left) side of an individual object regardless of the spatial position of that object [29,32,33]. Therefore, the investigation of multi-digit number processing may be specifically informative concerning the evaluation of an influence of object-based neglect, which has not been evaluated so far in the context of numerical cognition. When conceptualizing a multi-digit number as a coherent object, applying the logic of object-based symptoms of neglect would suggest specific neglect of the left part of a multi-digit number. In case of a two-digit number like 73 this neglect should manifest itself in possibly impaired processing of the leftward tens digit (i.e. 7). Importantly, comparable manifestations of object-based neglect have already been reported for the case of reading. On the one hand, in line with the notion of space-based neglect, neglect patients tend to omit whole words located on the contra-lesional side of a sheet of paper [34]. On the other hand, and of particular importance for the current study, these neglect patients also misread the beginnings of individual words because of omitting the first letters of a word (e.g., ‘word’ misread as ‘ord’, [35]). Such an error pattern is in line with the notion of object-based neglect (e.g., [29,32]). Transferring this phenomenon to the case of multi-digit numbers, we assume that object-based aspects of neglect should result in deficient processing of leftward digits within a specific number, such as the tens digit in a two-digit number.

In turn, such deficient processing of, for instance, the tens digit might then lead to impairments in processing tens and units complying with the place-value structure of the Arabic number system because not all digits at all positions to be integrated may be accessed or activated equally well. Yet, it is important to note that this does not necessarily mean that the decade digit is not processed at all. Quite to the contrary, we suggest that processing of the tens digit is less precise rather than omitted completely.

First evidence for such a very specific impairment of place-value processing came from the study by Hoeckner et al., [17]. In a number bisection task involving two-digit numbers only administered to neglect patients and two control groups, participants were presented with number triplets and asked whether the central number was also the arithmetical middle of the interval (e.g., 21_25_29) or not (e.g., (21_27_29). Performance of neglect patients differed from that of controls in two important respects: Apart from replicating influences of space-based neglect, these patients showed specific difficulties processing place-value information. In particular, they were particularly impaired for triplets crossing a decade boundary (e.g., 35_38_41) compared to triplets within the same decade (e.g., 32_35_38). Importantly, the finding that neglect patients were particularly impaired for triplets crossing a decade boundary is in line with the notion of symptoms of object-based neglect mentioned above (e.g., [29,32]) in number processing. One might hypothesize that processing of the left side of fixated objects, for instance the decade digit in a two-digit number, was specifically impaired in neglect patients and that therefore changes in the decade number were particularly difficult to process.

### The present study

Conceptualizing the observations of Hoeckner et al., [17] within the framework of space- and object-based neglect is not the only possible account. For both, the hypothesis referring to object-based as well as to space-based neglect, there are counter-arguments. As regards object-based neglect, one might argue that in the study of Hoeckner et al., [17] trials crossing a decade boundary were also generally much more difficult than trials not crossing that boundary, as documented by a high error rate of up to about 40% for the most difficult conditions (see also [36,37] for the influence of decade crossing in healthy adults). Therefore, Hoeckner et al., [17] may have observed a general difficulty effect.

As outlined above it was suggested recently that the apparent left-sided neglect in number bisection tasks may not necessarily reflect an isomorphism of physical and number space (as proposed by [11]; see [12] for a review) but may be driven by poor memorization of the initial items of the number sequences presented due to impaired working memory ([22,23]; for a review see [13]). For instance, van Dijck and colleagues [23] used tasks indeed relying heavily on working memory such as indicating the number midway between two verbally presented numbers in a number bisection task to investigate influences of neglect on number processing. Such working memory processes may be even more important for bisection tasks involving more complex and more difficult two-digit numbers [17]. Considering the issue of task difficulty, in the current study we pursued the issue of (impaired) representations of two-digit numbers in leftward neglect systematically employing the much easier number magnitude comparison task (cf. [38] with single-digit numbers), for which working memory influences should be less pronounced. If nevertheless differential effects can be found for neglect patients when performing such a task, this would also be informative with respect to the current debate on the origin of neglect-related deficits in number processing.

In our study a group of leftward neglect patients and two control groups (a patient control group with right-sided lesions but no neglect and a healthy control group) had to compare two-digit numbers to an internally memorized standard (i.e., 53 or 57). As we were particularly interested in effects of object-based neglect on place-value processing, the unit-decade compatibility effect, as observed in two-digit number magnitude comparison, seemed specifically informative. The unit-decade compatibility effect describes the observation that number pairs such as 32_47, in which separate comparisons of tens and units lead to the same decision (i.e., 3 < 4 and 2 < 7) RT and error rates are lower as compared to number pairs in which comparing tens and units separately yields incompatible decision biases (e.g., 37_52 for which 3 < 5 *but* 7 > 2) even though overall distance is held constant (i.e., 15 in both examples). As a consequence, the compatibility effect was interpreted to indicate separate and parallel processing of tens and units (e.g., [39-41]; [42] for a review) and thereby an influence of the place-value structure of the Arabic number system. When processes of place-value integration are specifically impaired in neglect patients, especially with regard to the tens digits, a specific modulation of the compatibility effect by neglect may be expected. As the compatibility effect is generally more pronounced for large (e.g., 71_39, with 9 > 1 by 8) as compared to small unit distances (e.g., 75_46, with 6 > 5 by 1, cf. [40]) two standards (i.e., 53 and 57) were used in the current study. This allowed for using an internal standard and unit distances up to 6 as compared to a standard of 55, where the maximum unit distance is 4 (see [39] for a more detailed discussion of this point). Thereby, we wanted to induce detection of the compatibility effect even in the case of small sample sizes and high variability of performance to be expected in neglect patients.

### Objectives and hypotheses

In the study by Hoeckner et al., [17] the number bisection task revealed strong differences between within- and between-decade items in neglect patients. From this pattern of results the authors concluded that neglect patients may have problems to mentally represent the decade digit’s magnitude, which are not due to perceptual impairments in neglect but due to impairments of the mental (spatial) representation of the presented two-digit numbers. Against this background the following more specific hypotheses can be derived.

First, associated with symptoms of object-based neglect, we hypothesized the unit-decade compatibility effect to be more pronounced in neglect patients as compared to controls. Because the tens digits of all two-digit numbers are located on the left, this compatibility effect could be due to possibly slower and less precise access to the magnitude of the tens’ digits in neglect patients. In turn, unimpaired access to the units should then exert a stronger influence on two-digit number processing, resulting in a more pronounced compatibility effect. Generally, the compatibility effect originates from interference between the (right-sided) *irrelevant* unit digit and the (left-sided) *relevant* decade digit, when these two digits are incompatible. Therefore, the stronger the focus on the irrelevant unit digit (for instance driven by neglecting the left-sided tens digit), the stronger the compatibility effect. Even more, in incompatible between-decade trials complete neglect of the tens digits would even generally lead to a wrong answer. Theoretically, such a finding would indicate that deficits in number processing and arithmetic may occur in neglect not only because the spatial representation of number magnitude along the MNL is impaired but also because the processing and integration of single digits of multi-digit numbers complying with the place-value structure of the Arabic number system may be impaired in neglect patients.

Second, our design, employing two separate internal standards, enabled us to contrast within-decade and between-decade comparisons even more directly than it had been possible in the study by Hoeckner et al., [17]. Comparisons with distances ±4, ±5, and ±6 were included for both between- and within-decade items (e.g., a distance of −5 corresponds to a between-decade comparison for the standard 53, i.e., 53_48, while it corresponds to a within-decade comparison for the standard 57, i.e., 57_52). Assuming the processing of the tens digits to be specifically impaired in neglect patients, we hypothesized that for the respective comparisons of within- and between-decade comparisons neglect patients should present with more difficulties for between- as compared to within-decade comparisons, because in the latter the tens digits are irrelevant. In summary, such a data pattern would provide first systematic evidence for the generalizability of the influence of object-based aspects of uni-lateral neglect to the processing of multi-digit numbers.

Despite these specific hypotheses we expected to replicate previous findings attributed to space-based symptoms of neglect, in particular, impaired processing of relatively smaller numbers associated with the left side of representational space. However, as the current manuscript focuses on the novel aspect of influences of object-based aspects of neglect on number processing, the interested reader is referred to Appendix A where the respective analyses and results are reported and discussed in detail.

## Methods

### Participants

Eighteen right-handed German-speaking volunteers took part in this study. Participants belonged to 3 groups of 6 persons each: (i) a patient group with right-sided lesions due to insult or haemorrhage of the right middle cerebral artery who suffered from left-sided neglect; (ii) a patient control group with right-sided lesions due to insult of the right middle cerebral artery but no clinical signs of neglect; and (iii) a healthy control group with no history of neurological or psychiatric illness. Participants were examined with the approval of the ethics committee of the province of Salzburg. The research was carried out in compliance with the Helsinki Declaration and written informed consent was obtained from the patients for publication of this report and any accompanying data or images. Groups were matched for age (mean age: 60.6 years; SD: 7.7 years), gender (4 males/2 females each), education and time post-lesion (see Table 1 for demographic and clinical details). In all cases of stroke (patient group and patient control group), lesions were confirmed by either MRI or CT. Visual field assessment showed no signs of hemianopia in any one of the participants. All participants had normal or corrected-to-normal vision.

**Table 1:** Demographic and clinical data of all participants

	**Sex**	**Age** (**yrs**)	**Education** (**yrs**)	**Time post**-**lesion** (**weeks**)	**Lesion etiology**	**Lesion site**	**Affected blood vessel**
Neglect group							
R.E.	Female	71	8	4	IS	RH	MCA
L.A.	Male	55	13	6	HS (BGH)	RH	MCA
R.A.	Female	63	8	5	HS (IP)	RH	MCA
K.W.	Male	49	13	6	IS	RH	MCA
P.A.	Male	70	11	13	IS	RH	MCA
F.J.	Male	54	11	7	HS (BGH)	RH	MCA
Patient control group							
C.K.	Female	68	7	6	IS	RH	MCA
S.G.	Male	55	13	10	IS	RH	MCA
G.G.	Female	66	10	15	IS	RH	MCA
J.G.	Male	52	17	38	IS	RH	MCA
F.E.	Male	68	17	11	HS	RH	SDH
P.T.	Male	54	12	5	IS	RH	MCA
Healthy control group							
L.I.	Female	72	10				
L.J.	Male	56	11				
D.M.	Female	60	12				
S.P.	Male	51	11				
L.G.	Male	71	8				
R.W.	Male	56	12				

RH - right hemisphere; IS - ischemic stroke; HS - hemorrhagic stroke; SDH - subdural hemorrhage; IP – intraparietal; MCA - middle cerebral artery; BGH - basal ganglia hemorrhage.

Please note that there are no significant differences for demographical and clinical variables. The neglect patient group does not differ from the control patient group regarding age [*t*(5) = 0.04; *p* = .97], education [*t*(5) = 1.14; *p* = .31] or time post-lesion [*t*(5) = 1.53; *p* = .19]. Equally, the neglect patient group does not differ from the healthy controls regarding age [*t*(5) = 0.13; *p* = .90] or education [*t*(5) < 0.01; *p* = 1]. Consequently, there is also no difference between the two control groups as regards age [*t*(5) = 0.11; *p* = .92] and education [*t*(5) = 1.24; *p* = .27].

The Neglect Test (NET, [43]; adapted German version of the Behavioural Inattention Test – BIT, [44] was used as a standardized neuropsychological neglect test-battery for diagnosing neglect in the patient group and for ruling out neglect in the patient control group. The NET includes 17 different tasks that can be allocated to the categories conventional subtests (e.g., line bisection, line and star cancellation, figure and shape copying, representational drawing) and behavioural subtests (e.g., picture scanning, menu reading, article reading, telling the time from analogue and digital clock faces, set the time). Moreover, to rule out degenerative cognitive symptoms, the SIDAM (Structured Interview for the Diagnosis of dementia of the Alzheimer type, Multi-infarct dementia and dementias of other etiology [45]) was administered. The SIDAM is a questionnaire for diagnosing dementia according to international diagnostic guidelines (ICD-10, DSM-IV). This instrument includes simple questions and problems covering areas such as orientation, instantaneous recall, memory (short-, long-term), intellectual/cognitive abilities, verbal and numerical abilities, visuo-spatial abilities, aphasia and apraxia. Additionally, the SIDAM also includes the Mini-Mental State Examination (MMSE, [46]), a dementia screening. To further investigate the participants for unspecific performance differences, also the adjusted SIDAM scores were determined. The adjusted SIDAM scores do not include items possibly affected by neglect or mental arithmetic.

The participants’ numerical and mathematical abilities were further evaluated using the EC 301 R [47]. The EC 301 R is a cognitive neuropsychological test-battery for the assessment of calculation and number processing capabilities in brain-damaged patients (German adaptation of the EC 301 assessment battery for brain damaged adults, cf. [48]. It comprises the subtests: dot counting, free backward counting, number transcoding, mental arithmetic, array on a physical number line, number comparison (auditory as well as symbolically), multi-digit arithmetic (i.e., addition, subtraction and multiplication), as well as perceptual and contextual estimation (cf. [48]).

None of the participants showed clinical signs of dementia or degenerative processes and all had good to perfect numerical and mathematical abilities so that the EC 301 R showed no sign of acalculia in any one of the patients. Table 2 summarizes the test results for the NET, MMSE, SIDAM, and EC 301 R.

**Table 2:** **Scores of individual participants in the NET** (**Neglect Test**), **in the MMSE** (**MiniMental State Examination**), **in the SIDAM** (**Structured Interview for the Diagnosis of dementia of the Alzheimer type**, **Multiinfarct dementia and dementias of other etiology**), **in the adjusted SIDAM**, **and in the EC 301 R** (**German adaptation of the EC 301 assessment battery for brain damaged adults**[36])

	**NET**	**MMSE**	**SIDAM**	**adj**. **SIDAM**	**EC 301 R**
Neglect group					
R.E.	135.0/170	26/30	42/55^a^	37/45	94/135
L.A.	70.0/170	24/30	42/55^a^	37/45	115/135^b^
R.A.	64.0/170	24/30	40/55^a^	35/45	78/135^b^
K.W.	86.0/170	25/30	44/55^a^	40/45	84/135^b^
P.A.	115.0/170	25/30	39/55^a^	35/45	110/135
F.J.	100.5/170	25/30	41/55^a^	35/45	108/135
Patient control group					
C.K.	170.0/170	28/30	51/55	44/45	127/135
S.G.	169.5/170	29/30	54/55	44/45	135/135
G.G.	169.5/170	30/30	52/55	42/45	131/135
J.G.	169.5/170	30/30	53/55	43/45	131/135
F.E.	169.0/170	29/30	48/55	38/45	129/135
P.T.	165.5/170	29/30	49/55	39/45	105/135
Healthy control group					
L.I.		28/30	48/55	39/45	134/135
L.J.		30/30	53/55	43/45	135/135
D.M.		29/30	52/55	42/45	135/135
S.P.		28/30	51/55	42/45	125/135
L.G.		28/30	52/55	42/45	133/135
R.W.		30/30	55/55	45/45	132/135

a Due to neglect symptoms some items of the SIDAM could not be successfully processed (e.g., copying shapes).

b Due to neglect symptoms some items of the EC 301 R could not be successfully processed (e.g., counting dots).

Please note that the neglect group differs significantly from both control groups, while there are no significant differences between the control groups. In particular, the neglect patient group differs from the control patient group regarding NET [t(5) = 3.33; p < .05], MMSE [t(5) = 25.80; p < .001], the SIDAM [t(5) = 18.46; p < .001], adjusted SIDAM [t(5) = 5.38; p < .01] as well as the EC 301 R [t(5) = 5.02; p < .01]. Similarly, the neglect patient group differs from the healthy controls regarding MMSE [t(5) = 16.98; p < .001], the SIDAM [t(5) = 20.78; p < .001], adjusted SIDAM [t(5) = 7.89; p < .001] as well as the EC 301 R[t(5) = 8.79; p < .001]. However, there was no difference between the two control groups as regards MMSE, SIDAM, adj. SIDAM and EC 301 R [all t(5) < 1.38; p > .23].

### Stimuli and design

In the current number comparison experiment participants had to decide whether a visually presented two-digit number was larger or smaller than an internal standard (either 53 or 57, respectively). The stimulus set included numbers ranging from 31 to 79 presented in Arabic notation but did not incorporate multiples of ten (e.g., 70). Moreover - depending on the standard employed - either the number 53 or 57 was omitted. Probe numbers smaller or larger than the standard were balanced in frequency of occurrence as was the number of between-decade (e.g., 53_45) and within-decade trials (e.g., 53_58). This latter requirement was met by selective repetitions of within-decade stimuli. Altogether, 72 probe numbers had to be compared to either standard. Thirty-six probe numbers were between-decade stimuli, 18 smaller (range 31 to 49 excluding 40) and 18 larger than the standard (range 61 to 79 excluding 70). Additionally, 36 within-decade stimuli were presented, again 18 smaller and 18 larger than the standard (cf. [39]) for a detailed description of the stimulus set and results for healthy student participants). This set of 50% within- and 50% between-decade stimuli is suited to accomplish that both tens and unit digits were decisive for the overall decision in a balanced manner. This stimulus set prevents focusing on either the decade or unit digit to be a beneficial strategy (e.g., [49]; [42] for a review).

To attain this balanced stimulus set, for the standard 57, each number from 51 to 56 was presented 3 times, and 58 and 59 both 9 times. For the standard 53 we accordingly included 9 times both 51 and 52 and 3 times 54 to 59.

### Procedure

The experiment was run on a 1.6 GHz laptop and participants were seated approximately 60 cm in front of a 15” screen driven at a resolution of 1024 × 768 pixels. Both instruction and numbers were presented in white “Arial” font (size 48) against a black background. Prior to each trial a fixation cross was presented in the centre of the screen for 1000 msec. Then the probe number was presented for a maximum time of 15,000 msec or until a response was given, followed by an ISI of 4000 msec.

The “Alt Gr” and the “Menue” keys located next to each other at the bottom right hand corner of a standard QWERTZ-keyboard served as response buttons: white stickers with the letters “kl” for “smaller” (in German “kleiner”) and “gr” for “larger” (in German “größer”) served as response keys, respectively. All other keys were covered up with black cardboard. Participants were instructed to indicate their decision by pressing one of the two response buttons as fast and as accurately as possible.

The instruction was followed by 12 practice trials. To ensure that patients perceived the presented numbers correctly, all participants were asked to read the numbers of the first four practice trials aloud. All patients were able to do so without problems. Furthermore, participation in the critical experiment was only allowed when more than 2/3 (8 out of 12) of the practice trials were classified correctly. All participants were able to do so without mistakes. The study took place on two testing days, involving presentation of 2 blocks per day. Each block consisted of all 72 probe numbers for one of the two standards. Trial order was randomized for each participant. Half of the participants started with comparing the probe to the internal standard 53 while the other half started with the internal standard 57. On the second testing day, the two blocks were presented again in reversed order. Participants initiated each trial by pressing one of the response keys. After 9 trials participants had the opportunity to interrupt the experiment for a break. The experiment took approximately 15–25 min, depending on response latencies and how long participants rested between and within blocks.

## Results

Before turning to the results it is important to note that none of the participants showed clinical signs of dementia or degenerative processes and all exhibited good to perfect numerical and mathematical abilities. In particular, the EC 301 R showed no sign of acalculia in any one of the patients, apart from some perceptual difficulties in processing specific items that seemed to be associated with symptoms of neglect rather than a general number processing deficit. Thus, the clinical examination demonstrated that number processing was not impaired per se in neglect patients.

For statistical analyses RT and error data were used. Only RTs for correct responses were incorporated in the analyses. Furthermore, response latencies shorter than 200 ms were not considered and in a second step latencies outside the interval of +/−3 standard deviations around the individual mean were excluded. Exclusion of erroneous responses and trimming resulted in a total loss of 9.5 % of the data (see Table 3). Because stroke patients showed more variability in their RTs than healthy participants, a z-transformation on individual item RT was carried out to control for such inter-individual overall differences in RT level. For the z-transformation, mean RT and the corresponding standard deviation of all correctly answered items were calculated for each participant individually and used for standardization. Thus, mean transformed zRT was 0 with a standard deviation of 1. As a consequence all possible influences of neglect on participants' performance cannot be driven by differences in overall RT between the respective participant groups.

**Table 3:** **Participant**-**based mean values of reaction time** (**RT**) **and error rate** (**ER**) **for each group in the compatible and incompatible conditions** (**with standard deviations in parentheses**)

	**Compatible**		**Incompatible**	
	**Mean RT[ms]**	**Mean ER [%]**	**Mean RT[ms]**	**Mean ER [%]**
Neglect group (n = 6)	3134 (847)	6.3 (5.9)	3070 (864)	13.5 (13.3)
Patient control group (n = 6)	1166 (403)	1.0 (1.8)	1177 (426)	1.5 (3.1)
Healthy control group (n = 6)	921 (199)	1.0 (2.3)	930 (211)	1.0 (1.7)

We consistently report the results of non-parametric analyses in the main text body starting with zRT followed by error rates. The *p*-values reported reflect exact significance. To give the reader a full grip of the data, these results are complemented by the results of (1) participant-based as well as (2) item-based parametric analyses on mean zRT and mean arcsine-transformed error rates provided in Appendix B.

### Unit-decade compatibility effect

No compatible/incompatible distinction is possible for probes deviating from the standard by a multiple of ten (e.g., 43 vs. 53). Therefore, these probes were not considered in analyses addressing the compatibility effect. For the remaining stimuli overall distance would have been larger for compatible than for incompatible number pairs. Therefore, 6 selectively chosen compatible as well as incompatible probes were excluded based on the constraint of balancing overall distance for compatible and incompatible number pairs. Note that matched overall distance can only be achieved by excluding specific, not by randomly chosen probes. Such an adjustment is mandatory because otherwise compatibility effects and distance effects are confounded. Due to this adjustment, incompatible probes 44, 45, 46, 47, 48, 49, as well as compatible probes 74, 75, 76, 77, 78, 79, were excluded in the standard 53 condition. Probes 31, 32, 33, 34, 35, 36 (compatible) and 61, 62, 63, 64, 65, 66 (incompatible) were omitted from the analysis for standard 57. In Appendix B, the impact of problem size was addressed additionally in an item-based parametric ANCOVA because it is not possible to match both overall distance and problem size [defined as 0.5 * (standard + probe)].

#### zRT

There was no significant main effect of participant group or a significant difference between the compatibility effects between participant groups on zRT as indicated by the Jonkheere-Terpstra test for monotone trend in group medians (both *p* > .15) nor a main effect of compatibility (*p* > .30, evaluated by the Wilcoxon-signed-ranks test in any of the respective participant groups).

#### Error rates

**Figure 1:**
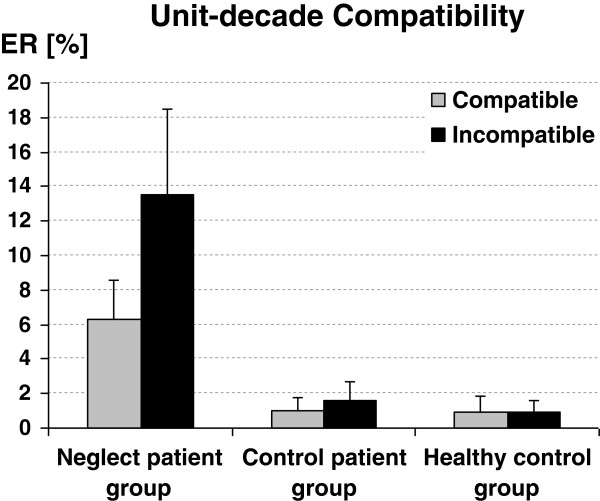
**Unit**-**decade compatibility effects.** The compatibility effects (i.e., error rate for incompatible and error rate for compatible number pairs) depicted separately for neglect patients, non-neglect control patients, and healthy controls. Please note that the compatibility effect can only be assessed for between-decade comparisons. Error bars indicate 1 SEM.

The Jonckheere-Terpstra test did not reveal significant group differences for overall error rate (*p* = .09; nor did the comparable Kruskal-Wallis test for the test against the less specific alternative hypothesis of at least one median difference between two of the three groups, *p* = .08), indicating that neglect patients did not commit more errors than the control groups. The Wilcoxon-signed-ranks test revealed a significant unit-decade compatibility effect for neglect patients (*p* < .05), but neither for the patient (*p* = .42) nor the healthy control group (*p* = .27). Neglect patients committed 13.5% errors in incompatible trials but only 6.3% errors in compatible trials (see Figure 1). Importantly, the Jonckheere-Terpstra test revealed that the compatibility effect differed reliably between the three groups (*p* < .05). Closer inspection by the Mann–Whitney-U test for the comparison of only two independent groups indicated that the difference in the compatibility effect was significant between neglect patients and healthy controls (*p* < .05, Z = 2.33) and marginally significant between neglect patients and patient controls (*p* = .06, Z = 1.9), whereas, it did not differ between the two control groups (*p* = .87, Z = 0.4).

Importantly, these results were substantiated by parametric testing as reported in Appendix B.

### Difference between within- and between-decade comparisons

**Figure 2:**
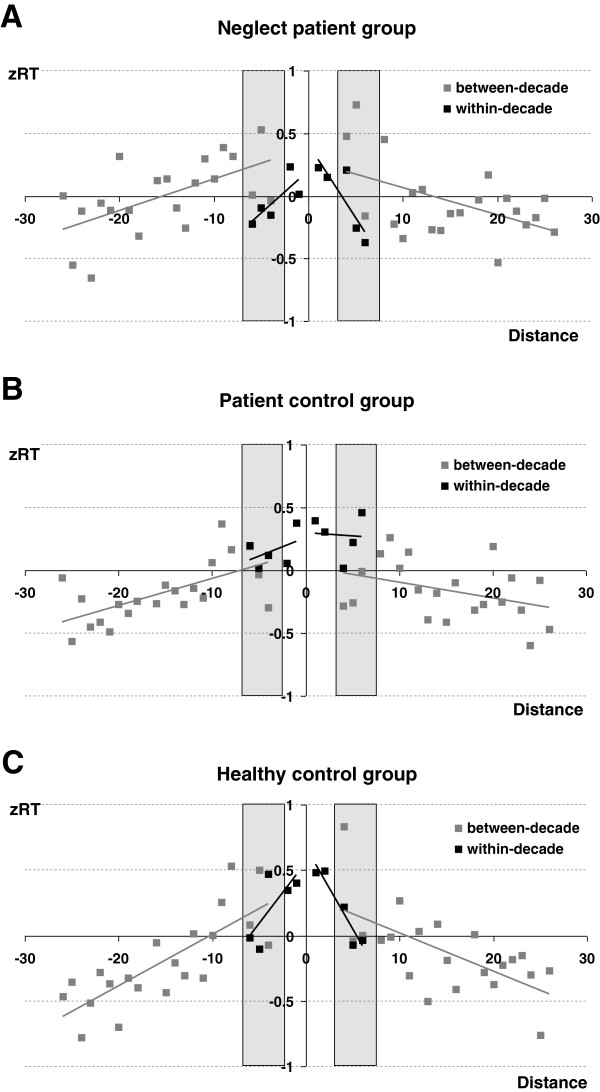
**The numerical distance effect.** z-transformed RT separately for between- (grey squares) and within-decade items(black squares). The x-axis indicates the numerical distance between probe and standard with negative values indicating probes smaller than the standard. Grey boxes highlight the distances ± 4, ± 5, and ± 6 that can be contrasted directly for between- and within-decade items as they were measured redundantly for both item types. **Panel A** gives the results for neglect patients. **Panel B** for the patient control group, and **Panel C** for the healthy control group.

We hypothesized that there might be specific differences in the performance of neglect patients when comparing between- and within-decade items. Importantly, our design with two separate internal standards enabled us to directly contrast within-decade and between-decade comparisons for the same distance (see the grey boxes in Figure 2A–C). In particular the distances ± 4, ± 5, and ± 6 were considered for both between- and within-decade items (e.g., a distance of −5 corresponds to a between-decade comparison for the standard 53, i.e., 53_48, while it corresponds to a within-decade comparison for the standard 57, i.e., 57_52).

#### zRT

The Jonckheere-Terpstra test on the comparison of within/between-decade items did not indicate a significant monotone trend in group medians for zRT (*p* = .47). Furthermore, the Wilcoxon-signed-ranks test regarding zRT revealed a main effect of within/between-decade processing for both, the neglect patients (*p* < .05) and for the control patients (*p* < .05) but not for the healthy controls (*p* = .17). However, while neglect patients responded 338 ms faster for within-decade comparisons, control patients showed an opposing pattern: between-decade items were responded to 15 ms faster than within-decade items. The Jonckheere-Terpstra test did not reveal a significant monotone trend in differences in within/between-decade processing (*p* = .19), whereas the comparable Kruskal-Wallis test for the test against the less specific alternative hypothesis of at least one median difference between two of the three groups revealed significant differences (*p* < .01). At closer inspection the Mann–Whitney-U test for the comparison of only two independent groups indicated that the difference in within/between-decade processing was significant only when comparing the neglect group and the patient control group (*p* < .01, Z = 2.88), but not when comparing neglect patients and the healthy control group (*p* = .12, Z = 1.6) or the two control groups (*p* = .07, Z = 1.9).

#### Error rates

The Jonckheere-Terpstra test did not reveal a significant main effect of group for the overall error rate (p = .42), indicating that neglect patients did not commit more errors in general due to more cognitive impairment. Furthermore, the Wilcoxon-signed-ranks test for error rates revealed a significant within/between decade effect for the neglect patients (*p* < .05), but not for the patient control group (*p* > .99) or the healthy control group (*p* > .99). Neglect patients committed 16.6% errors in between-decade items and only 8.8% in within-decade items Importantly, the Jonckheere-Terpstra test revealed the difference between within/between-decade comparisons to differ significantly across participant groups (*p* < .05). At closer inspection the Mann–Whitney-U test for the comparison of only two independent groups indicated that the difference between within/between-decade comparisons was significant for the neglect group and the healthy controls (*p* < .05, Z = 2.38), but not for neglect patients and the patient control group (*p* = .10, Z = 1.5) or the two control groups (*p* = .67, Z = 0.8).

Again, these results were substantiated by parametric testing (see Appendix B).

## Discussion

The current study set off to evaluate the potential impact of object-based neglect on numerical cognition. Conceptualizing two-digit numbers as an object, symptoms of object-based neglect should result in specifically impaired processing of the leftward tens digit of two-digit numbers. We hypothesized that unit-decade compatibility effects should be increased for neglect patients because of more pronounced interference caused by the units, when the impact of processing the tens is reduced. Second, we also expected specific differences for neglect patients when comparing the processing of selected within- and between-decade items with numerical distance held constant. Apart from these particular hypotheses with respect to an influence of object-based neglect, we also expected to replicate previous findings concerning space-based impairments due to neglect. Importantly, the present data were informative on all of these aspects. However, as an impact of space-based neglect (such as impaired processing of relatively smaller numbers, which we were able to replicate for two-digit numbers) were not at the heart of the current study, the interested reader is referred to the Appendix for a detailed description and discussion of the observations regarding space-based impairments. In the following, we will focus on the results concerning the impact of object-based neglect.

In line with our expectation of object-based neglect we observed the unit-decade compatibility effect to be more pronounced in neglect patients as compared to controls. As the unit-decade compatibility effect reflects unit-based interference on the overall decision process, the larger compatibility effect may represent the net effect of less intense processing of the tens and in turn relatively more dominant processing of the unit digit magnitudes in neglect patients. Second, with regard to the differentiation between the processing of within- vs. between-decade comparison, the results were also in line with our hypothesis of a specific impact of object-based neglect. Within-decade comparisons were indeed faster as compared to between-decade comparisons in neglect patients, but not so in controls.

Thus, the joint consideration of these patterns of results indicates that our data relate directly to previous findings on object-based neglect in reading research with particular neglect of the left part of to-be-read words, as already outlined in the introduction. Moreover, this pattern of results is particularly revealing for our understanding of how neglect impairs multi-digit number processing in a differential manner for space-based vs. object-based aspects of neglect.

### Object-based neglect impairments of two-digit number processing

As already described above we aimed at investigating impairments due to object-based neglect in number processing. Considering a two-digit number as an object, the notion of object-based neglect implies specific difficulties representing the left part of this object, i.e. the tens digit. The current data provide clear evidence that there were indeed particular difficulties for processing the tens digits. As reported above, neglect patients did show faster responses to within-decade comparisons when compared to between-decade comparisons with the same distance to the standard. Thus, neglect patients did not benefit from the fact that in between-decade comparisons tens and units differed between the to-be-compared numbers (as did the healthy control group). Instead, they seemed to benefit from a shared tens digit for probe and standard. One might hypothesize that processing of the left side of fixated objects, for instance the tens digit in a two-digit number, was specifically attenuated in neglect patients. This decade digit was irrelevant in within-decade comparisons (e.g., 52 vs. 53). Therefore, within-decade comparisons may have been considerably easier to process for neglect patient compared to between-decade comparison, because changes in the decade number, which were particularly difficult to process deeply, were negligible for the overall magnitude comparison.

Similarly, considering modulation of the unit-decade compatibility effect in case of neglect allows for new insights into the interrelation of number processing and neglect, which are not possible when using single-digit stimuli. In particular, we suggest that the observation of a unit-decade compatibility effect for neglect patients, while not observed in controls, can be assumed to reflect the influence of symptoms of object-based neglect on number processing as well. Because the decade digit of a two-digit number is the more leftward digit, object-based neglect for a two-digit number object should lead to more prominent processing of the more rightward unit digit. In turn, such deeper processing of the unit digit should result in a more pronounced unit-decade compatibility effect in neglect patients, which is exactly the pattern of results observed in the current study. Thereby, the current data are the first to provide clear evidence for impairments in multi-digit number processing due to symptoms of object-based neglect.

Yet, because the unit-decade compatibility effect is probably due to a conflict between the outcome of separate and parallel processing of tens and units [50], one should also consider the possibility that the effect may also be larger because of problems in executive control. However, brain imaging and TMS data concerning the potential locus of the unit-decade compatibility effect revealed that the unit-decade compatibility effect was correlated with neural activation in parietal regions and not in frontal areas, usually associated with executive control [37,51]. Moreover, TMS disruption of parietal processing has been proven to induce an increased unit-decade compatibility effect interpreted as a deficit in place-value integration. Thus, although we cannot exclude that impaired executive functions in neglect patients may also have added to the unit-decade compatibility effect, we are confident that the compatibility effect is mainly due to parietal processing of numerical information and not due to frontal processes of executive functions.

Taken together, our findings suggest that both object-based and space-based neglect affect number processing capabilities of neglect patients. Space-based neglect of representational number space seems to lead to impaired processing of the left side of the number line also for two-digit numbers (see Appendix A for details, see also [17]), whereas object-based neglect leads to impaired (reduced) processing of the left side of the object, i.e. means, the tens digit, when considering a two-digit number as one object. Consequently, the present study indicates that deficits in number processing and arithmetic may occur in neglect not only because access to the MNL representation of number magnitude is impaired, but also because single digits constituting a multi-digit number may not be appropriately integrated in the place-value structure of the Arabic number system due to object-based symptoms of neglect. Thus, neglect not only impairs the magnitude representation of two-digit numbers in general, but also specifically impairs the integration of the magnitude representation of tens and units in multi-digit number processing. At a broader level, this finding corroborates the notion that strategic effects, such as focusing attention primarily on the unit digits, influences multi-digit number processing. So far, such strategic effects have been evoked by the manipulation of stimulus sets, e.g., it was shown that the compatibility effect in number magnitude comparison increases with the number of within-decade items. An increase of within-decade comparisons leads to more prominent processing of the unit digits, because they become more and more salient for the decision as the proportion of within-decade items increases (e.g., [49,50]; see [42] for a review). The present study revealed consistent findings for the case of patient studies. Due to object-based unilateral neglect of the leftward tens digit, processing the unit digit may become more salient, so that the compatibility effect increases in turn.

### Limitations of the current study

In the present study we investigated the influence of object-based neglect symptoms on numerical cognition. Yet, it needs to be acknowledged that we did not systematically assess the degree of space- and object-based neglect when recruiting the patient sample. In our sample of patients indications of additional object-based neglect might have been appraised in the general neuropsychological assessment on the basis of standard clinical tests such as flower drawing. However, there was definitely no patient included who presented object-based neglect exclusively. In the majority of cases, aspects of object-based neglect may complement more obvious aspects of space-based neglect (for a review see [27]). Fink et al., [52] provided even evidence that the parietal areas implicated in object-based and space-based neglect reflect activation of a common attentional network (see also [53]). However, the idea that object-based neglect and space-based neglect recruit a common attentional network requires some caution. Studies on neglect patients showed that object-centred neglect is specifically associated with damage of middle occipital gyrus and posterior temporal cortex (see [31,54-58]).

Finally, there is also single-case evidence that space-based and object-based neglect may dissociate in neglect patients [59] or, in very rare cases, that object-based neglect even occurs without spatial neglect [60]. Therefore, we suggest it to be desirable for future studies to have the degree and possible dissociations between object-based and space-based neglect examined more systematically, for instance, using the Ota's search task [61] or the modified Ogden Scene test [62].

Moreover, we are well aware of the fact that conclusions drawn from heterogeneous patient samples should generally be interpreted with care, especially as neglect patients usually present quite wide-spread cognitive impairments including working memory deficits. As outlined in the introduction, there is an ongoing debate on how far neglect-related deficits in neglect may result from reduced working memory capacity (e.g., [23]). As regards working memory influences on the task used in the current study, the views in the literature are far from being conclusive. Of two studies evaluating working memory influences on the unit-decade compatibility effect one found no influence [63], while the other reported an interrelation between compatibility and some, but by far not all measures of working memory employed in the respective study [64]. However, we are confident that the results of the present study are not due to global effects of overall task difficulty or group differences with respect to working memory capacity, MMSE or SIDAM scores in neglect patients. For both effects of interest this claim is corroborated by the fact that our non-parametric tests revealed no overall differences between participant groups with regard to error rates. Additionally, as regards RT data, it has to be noted that all results reported refer to the analysis of z-transformed RT to control for influences of group differences in overall RT. Therefore, the differential effects observed should not be driven by group differences in overall RT. Finally, the results for the within/between decade comparison effect seem to be too specific to be due to a global effect of poor working memory. If this would have been the case, then the difference between neglect patients and controls should diminish for within-decade items (assuming that demands on working memory are lower for these items). However, neglect patients should *not* show a *reversed* pattern, which even contradicts previous results regarding the influence of the within/between-decade distinction on RT (e.g., [65]): neglect patients were significantly faster for within-decade items, while control patients showed an opposing pattern. This opposing pattern of results indicates that we in fact face differential rather than global difficulty effects. Nevertheless, for future studies it would be highly desirable to systematically assess working memory capacity to further investigate influences of neglect-related working memory impairments on numerical cognition.

### Perspectives

As outlined above, our data suggest that neglect impairs number processing of two-digit numbers in a very specific way: The relative neglect of the tens position in two-digit numbers in neglect patients may also be important for processes underlying mental arithmetic. For instance, addition problems requiring a carry operation (e.g., 38 + 26) are usually more difficult to perform than addition problems not requiring a carry (21 + 43; e.g., [66,67]). The same holds true for subtraction problems, which require borrowing (64 – 26) compared to problems that require no borrowing (64 – 21, e.g., [68]). Basically, the necessity of a carry / borrowing operation is determined by the unit digits of the operands in the respective addition / subtraction problem: For addition problems, whenever the sum of the unit digits of two operands is equal to or larger than 10 (38 + 26; unit sum 8 + 6 = 14), a carry operation is mandatory. For subtraction, a borrowing operation is required whenever the difference of the unit digits of two operands is smaller than 0 (64 – 38; unit sum 4 – 8 = −4). Generalizing our conclusion of a stronger influence of the units in case of neglect to the case of two-digit addition / subtraction we would predict a very specific pattern of results for neglect patients in mental addition / subtraction. In carry problems, the decade digit of the unit sum of the summands has to be carried (leftwards) to the decade position of the result to compute the correct solution. In borrowing problems, neglect patients would have to borrow from the neglected (left) part of the stimulus. In line with our argument for the stronger compatibility effect, symptoms of object-based neglect should result in particular difficulties for both the processing of carry and borrow problems in neglect patients because in both cases specific reference to the neglected tens digit is necessary. Moreover, at a more general level, considering previous findings on the effects of space-based neglect one may expect that subtraction should be relatively more impaired than addition because subtraction reflects a leftward movement on the MNL (e.g., [69]) and thus into the neglected part of number space. So far, these predictions remain speculative and should be investigated in future studies concerning the impact of space-based and object-based neglect on number processing.

## Conclusion

The data pattern of the current study provides converging evidence for generalizability of the influence of uni-lateral neglect to the processing of numbers beyond the single-digit number range. Moreover, as a consequence of using two-digit stimuli, we were able to apply the distinction of space-based and object-based neglect to the case of numerical cognition with particular interest in the impact of object-based neglect. Faster processing of within- as compared to between-decade items (with numerical distance held constant) and an enhanced unit-decade compatibility effect indexed more intense processing of the rightward unit digit within the object of a two-digit number. Our data corroborated the notion of object-based neglect for multi-digit numbers to affect number processing (and in particular, processes of place-value integration) in neglect patients. For future research, it might be informative to evaluate the generalizability of the space-based versus object-based neglect in mental arithmetic.

## Appendix A

### Influences of space-based neglect

#### Hypothesis

We hypothesized to replicate previous findings attributed to space-based symptoms of neglect. In particular, we expected impaired processing of relatively smaller numbers associated with the left side of representational space. This has previously been shown for single-digit numbers suggesting a specific form of space-based neglect for the left side of the MNL (e.g., [38]). Accordingly, we expected that neglect patients - in contrast to a control group - would not benefit from relatively smaller problem size, i.e., from the number to-be-compared being smaller than the internal standard (see [70] for a review). In particular, in neglect patients the problem size effect was expected to be reversed as compared to controls.

#### Results

The RT problem size effect was evaluated using a 3 × 2 ANOVA comprising the between-subject factor group (neglect patients, patient control group, healthy control group), and the within-subject factor problem size (smaller vs. larger than standard). Please note that there were no significant results in the error analyses, possibly due to the fact that overall error rate was very low.

The ANOVA indicated no reliable main effects of either group *F* (2, 15) = 1.38, *p* = .28] or problem size *F* (1, 15) < 1], nor was the interaction of group and problem size significant *F* (2, 15) = 1.68, *p* = .22]. Because we had a specific hypothesis regarding differences of the problem size effect between the participant groups, the problem size effect was inspected more closely by a procedure identical to the one used for the distance effect. As discussed by Hager [71], the overall ANOVA interaction term may be too conservative to test for a specific interaction hypothesis, in particular for factors involving more than two levels. Therefore, we first evaluated whether the two control groups differed with regard to the problem size effect by a similar ANOVA comprising only RH control patients and healthy controls. As indicated by the non-significant interaction of problem size and group, the two control groups did not differ with respect to the problem size effect and thus their data was pooled *F*(1, 11) = .01, *p* = .92]. Importantly, as indicated by a final ANOVA comprising neglect patients and the pooled control groups, the problem size effect for neglect patients differed from that for the pooled control group in the expected direction meaning that neglect patients did not benefit from a smaller problem size *t*(16) = 1.87, *p* < .05]. In contrast, neglect patients even experienced disadvantages (RT prolonged by 116 ms on average) compared to the advantage for the control groups (RT reduced by 23 ms on average) whenever they had to classify probes as smaller than the standard. Importantly, the group differences regarding the problem size effect can not be explained by systematic differences in impaired cognitive processing, because an additional ANCOVA incorporating the adjusted SIDAM-value as covariate substantiated the group differences.

#### Discussion

As regards the problem size effect we found a typical pattern of space-based neglect. In contrast to controls, neglect patients did not benefit from a relatively smaller problem size. Instead, a ‘relatively smaller problem size’ (i.e., the probe being smaller than the internal standard) was associated with longer instead of faster responses. Thereby, these data provide converging evidence for the claim that neglect affects the left side of representational number space (e.g., [11]).

## Appendix B

### Compatibility effect

#### Parametric participant-based

The non-parametric results were corroborated by parametric tests: Similar to the item-based ANOVA, there were no significant main effects or interactions when analyzing zRT. Participant-based evaluation of arcsince-transformed error rate revealed significant main effects of group [*F*(2,15) = 7.5, *p* < .01] and unit-decade compatibility [*F*(1,15) = 8.4, *p* < .05]: Neglect patients committed significantly more errors (9.9%) than patient controls (1.3%) or healthy controls (1.0%). Participants committed significantly more errors in incompatible (5.4%) than in compatible items (2.8%). Bonferroni-corrected pairwise comparisons indicated that neglect patients exhibited a reliably higher error rate than both control groups (both *p* < .01), while patient controls and healthy controls did not differ from each other (*p* = .89). Additionally, the interaction of group and compatibility was significant [*F*(2, 15) = 4.9, *p* < .05]. To evaluate this pattern of results in more detail, we first ran an ANOVA over mean arcsine-transformed error rates with compatibility as repeated measures factor incorporating only the two control groups. Both control groups did not differ regarding the compatibility effect as indicated by the non-significant interaction of compatibility and group [F(1, 10) < 1; *p* = .98]. In contrast, the respective ANOVA incorporating the neglect patients and the patient control group revealed a reliable interaction of compatibility and group [F(1,10) = 5.3, *p* < .05], as did the ANOVA including neglect patients and healthy controls [*F*(1,10) = 5.5, *p* < .05]. This indicated the compatibility effect to be more pronounced for the neglect group (7.2% errors) as compared to both the patient (0.5% errors) as well as the healthy control group (0.1% errors). Finally, Bonferroni-corrected *t*-tests for each of the three groups individually indicated that the unit-decade compatibility effect was significant only for the neglect group [*t*(5) = 2.7; *p* < .05], but neither for the patient control [*t*(5) = 0.73; *p* = .50], nor for the healthy control group [*t*(5) = 0.79; *p* = .46].

#### Parametric item-based

The unit-decade compatibility effect was analyzed using a univariate 3 × 2 ANCOVA over mean zRT and mean arcsine-transformed error rates of all between-decade stimuli comprising the between-subject factor ‘group’ (neglect patients, patient control group, healthy control group), and ‘compatibility’ (e.g., 43_57 vs. 49_57). The ANCOVA was conducted over items because problem size can only be computed for trials but not participants.

The item-based parametric results again corroborated the non-parametric tests: There were no significant main effects or interactions when comparing zRT. Nevertheless, evaluation of arcsine-transformed error rates revealed significant main effects of group *F*(2,130) = 26.8, *p* < .001] and of unit-decade compatibility *F*(1,131) = 4.1, *p* < .05]: Neglect patients committed significantly more errors (9.1%) than patient controls (1.9%) or healthy controls (0.8%). Bonferroni-corrected pairwise comparisons indicated that the neglect patients exhibited a reliably higher error rate than both control groups (both *p* < .001), while patient controls and healthy controls did not differ from each other as regards error rates (*p* = .20). Moreover, in unit-decade incompatible trials participants committed generally more errors than in compatible trials (5.1% vs. 2.8%). The interaction of group and compatibility was not significant *F*(2,130) = 2.3, *p* = .10]. However, as demonstrated in detail by Hager [71], an AN(C)OVA interaction test may be too conservative to test specific interaction hypotheses, and in particular so, when a factor has more than two levels. For instance, when two groups do not differ with regard to a particular interaction (like controls and RH patients without neglect in the present study) the overall interaction may not turn out to be significant. To test the latter hypothesis, in a first step we repeated the ANCOVA including only the two control groups under the grouping factor. In line with our hypothesis, these groups did not differ regarding the compatibility effect as indicated by the non-significant group by compatibility interaction [F(1, 86) < 1; *p* = .56] even at a liberal type-I error level (of 10 or even 20%) to guard against a large type-II error level. In contrast, the respective ANCOVA incorporating the neglect patients and the patient control group revealed a reliable interaction of compatibility and group [F(1,86) = 3.9, *p* < .05], as did the ANCOVA including neglect patients and healthy controls *F*(1,86) = 4.0, *p* < .05]. Therefore, we pooled the data (see [67]) and conducted the respective ANCOVA comprising the neglect patients and the pooled control groups. Importantly, the group by compatibility comparison interaction was reliable [F(1, 130) = 4.4; *p* < .05] indicating that the compatibility effect was more pronounced for the neglect group (6% errors) as compared to the pooled control groups (0.4% errors). Finally, separate univariate ANCOVAs for each of the three groups individually, with compatibility as repeated measures factor and problem size as a covariate indicated that the unit-decade compatibility effect was significant only for the neglect group *F*(1, 43) = 4.4, *p* < .05] but neither for the patient control nor for the healthy control group [both *F*(1, 43) < 1].

### Within-between decade comparison effect

#### Parametric participant-based

##### zRT

Participant-based evaluation of zRT revealed significant main effects of group *F*(2,15) = 4.5, *p* < .05] and within/between-decade comparison *F*(1,15) = 16.7, *p* < .01]: Bonferroni-corrected pairwise comparisons indicated that the neglect patients responded more slowly than both control groups (both *p* < .05), while patient controls and healthy controls did not differ from each other (*p* = .71). The interaction between within/between-decade comparison and participant group was not significant *F*(2, 15) = 2.3; *p* = .13]. However, as the two control groups did not differ regarding the variable of interest [F(1, 10) < 1; *p* = .86] even at a liberal type-I error level (of 10 or even 20%) to guard against a large type-II error level, we pooled the data (see [71]) and conducted the respective ANOVA comprising the neglect and the pooled control groups. Importantly, the group by within/between-decade comparison interaction was reliable [F(1, 16) = 4.9; *p* < .05] indicating that the difference for within- and between-decade comparisons was more pronounced for neglect patients: while for the pooled control group responses to within-decade comparisons were 15 ms slower than to between-decade comparisons, this pattern was reversed for neglect patients who exhibited responses 338 ms faster for within-decade comparisons.

##### Error rates

The participant-based ANOVA on the arcsine-transformed error rates revealed a significant main effect of group [*F*(2,15) = 6.8, *p* < .01] and of within/between-decade comparison [*F*(1,15) = 1.7, *p* = .21]. Bonferroni-corrected pairwise comparisons indicated that the neglect patients (14.2%) had a reliably higher error rate than both control groups (both *p* < .01), while patient controls (7.0%) and healthy controls (7.1%) did not differ from each other (*p* = .92). The interaction of group and within/between decade comparison was not reliable [*F*(2,15) = 1.7, *p* = .21]. Though the two control groups did not differ regarding within/between decade comparison, [F(1, 10) < 1; *p* = .57] pooling of the control groups did only lead to a marginally significant group by within/between-decade comparison interaction [*F*(1, 16) = 3.4; *p* = .08].

#### Parametric item-based

A repeated measures item-based ANOVA with within/between decade comparison as within- and group membership as between-subject factor (i.e., neglect patients, healthy controls, control patients) was conducted to evaluate group differences in the processing of within and between-decade pairs.

##### zRT

When considering all three participant groups, neither the main effect of group [*F*(2, 15) = 1.17; *p* = .30] nor the main effect of within/between decade comparison [*F*(1, 15) < 1; *p* = .59] was significant. However, the interaction between within/between-decade comparison and group was reliable [*F*(2, 15) = 6.51; *p* < .01]. In order to break down this interaction, we ran separate ANOVAs, first incorporating the neglect and the patient control group only. These two groups differed reliable regarding the within/between-decade comparison effect as indicated by the significant interaction of within/between-decade comparison and group F(1,10) = 18.7; *p* < .01]. In particular, neglect patients responded 342 ms faster on average in within-decade items as compared to between-decade items, while this pattern was reversed for the patient control group (152 ms). In contrast, the interaction of within/between-decade comparison and group was neither reliable for neglect patients and healthy controls [*F*(1,1 0) = 1.4, *p* = .26], nor for the two control groups [*F*(1, 10) = 4.8, *p* = .07].

##### Error rates

When considering all participant groups, both the main effect of within/between decade [*F*(1, 15) = 5.2; *p* < .05] as well as the main effect of group were significant [*F*(2, 15) = 17.5; *p* < .001]. Pairwise comparisons revealed that the neglect group committed significantly more errors (12.7%, *p* < .001) than both, the patient control group (2.5%) as well as the healthy control group (2.3%), while the two control groups did not differ. Unexpectedly, participants committed significantly more errors in between-decade items (7.9%) than in within-decade items (3.9%). However, according to our hypotheses this effect should be driven by the fact that neglect patients in particular committed more errors for between (16.7%) than for within-decade comparisons (8.8%). The marginally significant interaction between within/between decade comparison and group lent first support to this assumption [*F*(2, 15) = 2.8; *p* = .07]. To evaluate this interaction in more detail, we again ran separate ANOVAs. As for zRT, the ANOVA incorporating the neglect and the patient control group revealed a reliable interaction of within/between decade comparison and group [*F*(1,10) = 4.1, *p* < .05] indicating the difference for within- and between-decade comparisons to be more pronounced for neglect patients (7.9% vs. 0.5% errors). As expected, neglect patients committed significantly more errors in between- as compared to within-decade comparisons [*t*(5) = 10.0; *p* < .001; 16.7% vs. 8.8% errors, respectively]. Moreover, similar to the results for zRT the test for an interaction of within/between-decade comparison and group was neither significant for neglect patients and healthy controls [F(1,10) = 1.8; *p* = .21] nor the two control groups [F(1,10) = 1.6; *p* = .23],

## Competing interests

The authors declare that they have no competing interests.

## Authors’ contributions

HZ, GW, KM and HCN conceived the study. All authors participated in its design. DZ, CH and AG performed data collection. DZ, GW, EK and KM performed data processing and statistical analyses. EK and KM drafted the manuscript. All authors contributed to the interpretation of the data. All authors read and approved the final manuscript.

## Authors’ information

Elise Klein and Korbinian Moeller contributed equally to this manuscript and should therefore be considered shared first authors.

## References

[B1] Vallar G Robertson IH, Marshall JC The anatomical basis of spatial hemineglect in humans Unilateral neglect: Clinical and experimental studies 1993 Hillsdale, USA: Lawrence Erlbaum Associates 27 62

[B2] Mesulam MM Spatial attention and neglect: parietal, frontal and cingulate contributions to the mental representation and attentional targeting of salient extrapersonal events Philos Trans R Soc Lond B Biol Sci 1999 354 1325 1346 10.1098/rstb.1999.0482 10466154 PMC1692628

[B3] Karnath H-O New insights into the functions of the superior temporal cortex Nat Rev Neurosci 2001 2 568 576 11484000 10.1038/35086057

[B4] Kerkhoff G Spatial hemineglect in humans Prog Neurobiol 2000 63 1 27 11040416 10.1016/s0301-0082(00)00028-9

[B5] Halligan PW Fink GR Marshall JC Vallar G Spatial cognition: evidence from visual neglect Trends Cogn Sci 2003 3 125 133 12639694 10.1016/s1364-6613(03)00032-9

[B6] Goodale MA Milner AD Jakobson LS Carey DP Kinematic analysis of limb movements in neuropsychological research: Subtle deficits and recovery of function Can J Psychol 1990 44 180 195 2200594 10.1037/h0084245

[B7] Marshall J Halligan P When right goes left: an investigation of line bisection in a case of visual neglect Cortex 1989 25 503 515 2805737 10.1016/s0010-9452(89)80065-6

[B8] De Hevia MD Vallar G Girelli L Visualizing numbers in the mind's eye: The role of visuo-spatial processes in numerical abilities Neurosci Biobehav R 2008 32 8 1361 1372 10.1016/j.neubiorev.2008.05.015 18584868

[B9] Dehaene S Piazza M Pinel P Cohen L Three parietal circuits for number processing Cognitive Neuropsych 2003 20 487 506 10.1080/02643290244000239 20957581

[B10] Bueti D Walsh V The parietal cortex and the representation of time, space, number and other magnitudes Philos Trans R Soc B Biol Sci 2009 364 1831 1840 10.1098/rstb.2009.0028 PMC2685826 19487186

[B11] Zorzi M Priftis K Umiltà C Neglect disrupts the mental number line Nature 2002 417 138 139 10.1038/417138a 12000950

[B12] Umilta C Priftis K Zorzi M The spatial representation of numbers: evidence from neglect and pseudoneglect Exp Brain Res 2009 192 561 569 10.1007/s00221-008-1623-2 18985329

[B13] Rossetti Y Jacquin-Courtois S Aiello M Ishihara M Brozzoli C Doricchi F Dehaene S, Brannon E Neglect “Around the Clock”: Dissociating Number and Spatial Neglect in Right Brain Damage Space, Time and Number in the Brain 2011 London: Academic Press 149 173 10.1016/B978-0-12-385948-8.00011-6

[B14] Halligan PW Marshall JC How long is a piece of string? A study of line bisection in a case of visual neglect Cortex 1988 2 321 328 3416614 10.1016/s0010-9452(88)80040-6

[B15] Zorzi M Bonato M Treccani B Scalambrin G Marenzi R Priftis K Neglect impairs explicit processing of the mental number line Front Hum Neurosci 2012 6 125 22661935 10.3389/fnhum.2012.00125 PMC3356871

[B16] Doricchi F Guariglia P Gasparini M Tomaiuolo F Dissociation between physical and mental number line bisection in right hemisphere brain damage Nat Neurosci 2005 8 1663 1666 10.1038/nn1563 16261135

[B17] Hoeckner SH Zauner H Moeller K Wood G Haider C Gaßner A Nuerk HC Impairments of the mental number line for two-digit numbers in neglect Cortex 2008 44 429 438 10.1016/j.cortex.2007.09.001 18387575

[B18] Priftis K Zorzi M Meneghello F Marenzi R Umiltà C Explicit versus implicit processing of representational space in neglect: dissociations in accessing the mental number line J Cogn Neurosci 2006 18 680 688 10.1162/jocn.2006.18.4.680 16768369

[B19] Zorzi M Priftis K Meneghello F Marenzi R Umiltà C The spatial representation of numerical and non-numerical sequences: evidence from neglect Neuropsychologia 2006 44 1061 1067 10.1016/j.neuropsychologia.2005.10.025 16356515

[B20] Cappelletti M Freeman ED Cipolotti L The middle house or the middle floor: bisecting horizontal and vertical mental number lines in neglect Neuropsychologia 2007 45 2989 3000 10.1016/j.neuropsychologia.2007.05.014 17640687 PMC2567815

[B21] Zamarian L Egger C Delazer M The mental representation of ordered sequences in visual neglect Cortex 2007 43 542 550 10.1016/S0010-9452(08)70248-X 17624000

[B22] Fias W van Dijck JF Gevers W Dehaene S, Brannon EM How number is associated with space? The role of working memory Space, time and number in the brain: Searching for the foundations of mathematical thought 2011 Burlington, MA: Elsevier/Academic Press 133 148

[B23] Van Dijck JP Gevers W Lafosse C Doricchi F Fias W Non-spatial neglect for the mental number line Neuropsychologia 2011 49 9 2570 2583 10.1016/j.neuropsychologia.2011.05.005 21605574

[B24] Aiello M Jacquin-Courtois S Merola S Ottaviani T Tomaiuolo F Bueti D Rossetti Y Doricchi F No inherent left and right side in human 'mental number line': evidence from right brain damage Brain 2012 135 2492 2505 10.1093/brain/aws114 22577222

[B25] Driver J Halligan PW Can visual neglect operate in object-centred coordinates? An affirmative single-case study Cogn Neuropsych 1991 8 6 475 496 10.1080/02643299108253384

[B26] Marshall JC Halligan PW The yin and the yang of visuo-spatial neglect: A case study Neuropsychologia 1993 32 9 1037 1057 7991072 10.1016/0028-3932(94)90151-1

[B27] Walker R Spatial and object-based neglect. Review Neurocase 1995 1 371 383 10.1080/13554799508402381

[B28] Posner MI Orienting of attention Q J Exp Psychol 1980 32 1 3 25 10.1080/00335558008248231 7367577

[B29] Duncan J Selective attention and the organization of visual information J Exp Psychol Gen 1984 113 4 501 517 6240521 10.1037//0096-3445.113.4.501

[B30] Gainotti G Messerli P Tissot R Qualitative analysis of unilateral spatial neglect in relation to laterality of cerebral lesions J Neurol Neurosurg Psychiatry 1972 35 4 545 550 10.1136/jnnp.35.4.545 5049813 PMC494120

[B31] Karnath HO Mandler A Clavagnier S Object-based neglect varies with egocentric position J Cogn Neurosci 2011 23 10 2983 2993 10.1162/jocn_a_00005 21391769

[B32] Behrmann M Plaut DC The interaction of spatial reference frames and hierarchical object representations: evidence from figure copying in hemispatial neglect Cogn Affective Behav Neurosci 2001 1 307 329 10.3758/CABN.1.4.307 12467084

[B33] Karnath HO Niemeier M Task-dependent differences in the exploratory behaviour of patients with spatial neglect Neuropsychologia 2002 40 9 1577 1585 10.1016/S0028-3932(02)00020-9 11985839

[B34] Riddoch MJ Humphreys GW Willows DM, Kruk RS, Corcos EM Visual aspects of neglect dyslexia Visual Processes in Reading and Text Disabilities 1991 New York: LEA 111 136

[B35] Kinsbourne M Warrington EK A variety of reading disability associated with right hemisphere lesions J Neurol Neurosur PS 1962 25 339 344 10.1136/jnnp.25.4.339 PMC495484 14032919

[B36] Nuerk HC Weger U Willmes K A unit-decade compatibility effect in German number words Curr Psychol Lett Behavi Brain Cogn 2002 2 19 38

[B37] Wood G Nuerk HC Moeller K Geppert B Schnitker R Weber J Willmes K All for one but not one for all: How multiple number representations are recruited in one numerical task Brain Res 2008 1187 154 166 18022606 10.1016/j.brainres.2007.09.094

[B38] Vuilleumier P Ortigue S Brugger P The number space and neglect Cortex 2004 40 399 410 10.1016/S0010-9452(08)70134-5 15156797

[B39] Moeller K Nuerk HC Willmes K Internal magnitude representation is not holistic, either Eur J Cogn Psychol 2009 21 672 685 10.1080/09541440802311899

[B40] Nuerk HC Weger U Willmes K Decade breaks in the mental number line? Putting the tens and units back in different bins Cognition 2001 82 B25 B33 10.1016/S0010-0277(01)00142-1 11672709

[B41] Nuerk HC Willmes K On the magnitude representations of two-digit numbers Psychol Sci 2005 47 52 72

[B42] Nuerk HC Moeller K Klein E Willmes K Fischer MH Extending the mental number line - a review of multi-digit number processing J Psychol 2011 219 3 22

[B43] Fels M Geissner E Neglect-Test (NET) 1997 2 Göttingen: Hogrefe

[B44] Wilson BA Cockburn J Halligan P The Behavioural Inattention Test (BIT) 1987 Thurston: Thames Valley Test Company

[B45] Zaudig M Hiller W Geiselmann B Hansert E Linder G Mombour W Reischies FM Thora C SIDAM – Strukturiertes Interview für die Diagnose einer Demenz vom Alzheimer Typ, der Multiinfarkt- (oder vaskulären) Demenz und Demenzen anderer Ätiologie nach DSM-III-R, DSM-IV und ICD-10 1996 Göttingen: Hogrefe

[B46] Folstein MF Folstein SE McHugh PR Mini-Mental-Status-Test (MMST) 2000 Göttingen: Hogrefe

[B47] Claros Salinas D EC 301-R: Untersuchungsmaterial zu Störungen des Rechnens und der Zahlenverarbeitung 1994 Konstanz: Kliniken Schmieder

[B48] Deloche S Seron X Larroque C Magnien C Metz-Lutz MN Noël MN Riva I Schils JP Dordain M Ferrand I Baeta E Basso A Cipolotti L Claros Salinas D Howard D Gaillard F Goldenberg G Mazzucchi A Stachowiak F Tzavaras A Vendrell J Bergego C Pradat-Diehl P Calculation and number processing: assessment battery: role of demographic factors J Clin Exp Neuropsychol 1994 16 195 208 10.1080/01688639408402631 8021307

[B49] Mann A Moeller K Pixner S Kaufmann L Nuerk HC Attentional strategies in place–value integration: a longitudinal study on two-digit number comparison J Psychol 2011 219 42 49

[B50] Macizo P Herrera A Cognitive control in number processing: Evidence from the unit–decade compatibility effect Acta Psychol 2011 136 112 118 10.1016/j.actpsy.2010.10.008 21078509

[B51] Knops A Nuerk HC Sparing R Foltys H Willmes K On the functional role of human parietal cortex in number processing: How gender mediates the impact of a ‘virtual lesion’ induced by rTMS Neuropsychologia 2006 44 2270 2283 10.1016/j.neuropsychologia.2006.05.011 16828812

[B52] Fink GR Dolan RJ Halligan PW Marshall JC Frith CD Space-based and object-based visual attention: shared and specific neural domains Brain 1997 120 11 2013 2028 10.1093/brain/120.11.2013 9397018

[B53] Tipper SP Behrmann M Object-centered not scene-based visual neglect J Exp Psychol Hum Percept Perform 1996 22 1261 1278 8865621 10.1037//0096-1523.22.5.1261

[B54] Hillis AE Newhart M Heidler J Barker PB Herskovits EH Degaonkar M Anatomy of spatial attention: insights from perfusion imaging and hemispatial neglect in acute stroke J Neurosci 2005 25 12 3161 3167 10.1523/JNEUROSCI.4468-04.2005 15788773 PMC6725074

[B55] Grimsen C Hildebrandt H Fahle M Dissociation of egocentric and allocentric coding of space in visual search after right middle cerebral artery stroke Neuropsychologia 2008 46 3 902 914 10.1016/j.neuropsychologia.2007.11.028 18206963

[B56] Medina J Kannan V Pawlak MA Kleinman JT Newhart M Davis C Heidler-Gary JE Herskovits EH Hillis AE Neural substrates of visuospatial processing in distinct reference frames: evidence from unilateral spatial neglect J Cogn Neurosci 2009 21 11 2073 2084 10.1162/jocn.2008.21160 19016599 PMC2828044

[B57] Verdon V Schwartz S Lovblad KO Hauert CA Vuilleumier P Neuroanatomy of hemispatial neglect and its functional components: a study using voxel-based lesion-symptom mapping Brain 2010 133 3 880 894 10.1093/brain/awp305 20028714

[B58] Khurshid S Trupe LA Newhart M Davis C Molitoris JJ Medina J Leigh R Hillis AE Reperfusion of specific cortical areas is associated with improvement in distinct forms of hemispatial neglect Cortex 2012 48 5 530 539 10.1016/j.cortex.2011.01.003 21345430 PMC3125403

[B59] Humphreys GW Riddoch MJ Attention to within-object and between-object spatial representations: Multiple sites for visual selection Cogn Neuropsychol 1994 11 2 207 241 10.1080/02643299408251974

[B60] Walker R Findlay JM Saccadic eye movement programming in unilateral neglect Neuropsychologia 1996 34 6 493 508 10.1016/0028-3932(95)00156-5 8736563

[B61] Ota H Fujii T Suzuki K Fukatsu R Yamadori A Dissociation of bodycentered and stimulus centered representations in unilateral neglect Neurology 2001 57 2064 2069 11739827 10.1212/wnl.57.11.2064

[B62] Ogden JA Anterior-posterior interhemispheric differences in the loci of lesion producing visual hemineglect Brain Cogn 1985 4 59 75 10.1016/0278-2626(85)90054-5 4027055

[B63] Macizo P Herrera A Román P Martín MC Proficiency in a Second Language Influences the Processing of Number Words J Cogn Psychol 2011 23 915 921 10.1080/20445911.2011.586626

[B64] Macizo P Herrera A Working memory and two-digit number processing Memory 2011 19 8 941 955 10.1080/09658211.2011.614621 22059625

[B65] Verguts T De Moor W Two-digit comparison: decomposed, holistic, or hybrid? Exp Psychol 2005 52 3 195 200 10.1027/1618-3169.52.3.195 16076067

[B66] Klein E Moeller K Dressel K Domahs F Wood G Willmes K Nuerk HC To carry or not to carry – is this the question? Disentangling the carry effect in multi-digit addition Acta Psychol 2010 135 67 76 10.1016/j.actpsy.2010.06.002 20580340

[B67] Kong J Wang C Kwong K Vangel M Chuac E Gollub R The neural substrate of arithmetic operations and procedure complexity Cogn Brain Res 2005 22 397 405 10.1016/j.cogbrainres.2004.09.011 15722210

[B68] Brownell WA Borrowing in subtraction J Educ Res 1940 33 6 415 424

[B69] Knops A Thirion B Hubbard EM Michel V Dehaene S Recruitment of an area involved in eye movements during mental arithmetic Science 2009 324 1583 1585 10.1126/science.1171599 19423779

[B70] Zbrodoff NJ Logan GD Campbell JID What everyone finds: The problem-size effect Handbook of Mathematical Cognition 2005 New York, NY: Psychology Press 331 345

[B71] Hager W The examination of psychological hypotheses by planned contrasts referring to two-factor interactions in fixed-effects ANOVA Method Psychol Res Online 2002 7 49 77

